# Modeling Canine Hemangiosarcoma Progression Using Patient-Derived 2.5D Organoids and Orthotopic Xenografts

**DOI:** 10.3390/vetsci13090879

**Published:** 2026-08-27

**Authors:** Yishan Liu, Haru Yamamoto, Mohamed Elbadawy, Amira Abugomaa, Masahiro Kaneda, Yomogi Shiota, Tadashi Kondo, Tatsuya Usui, Kazuaki Sasaki

**Affiliations:** 1 Laboratory of Veterinary Pharmacology, Department of Veterinary Medicine, Faculty of Agriculture, Tokyo University of Agriculture and Technology, 3-5-8 Saiwai-Cho, Fuchu, Tokyo 183-8509, Japan; 2 AIRDEC Mini Co., Ltd., 1-2-36 Kajino-Cho, Koganei, Tokyo 184-0002, Japan; 3 Department of Pharmacology, Faculty of Veterinary Medicine, Benha University, Moshtohor, Toukh 13736, Egypt; 4 Faculty of Veterinary Medicine, Mansoura University, Mansoura 35516, Egypt; 5 Laboratory of Veterinary Anatomy, Department of Veterinary Medicine, Faculty of Agriculture, Tokyo University of Agriculture and Technology, 3-5-8 Saiwai-Cho, Fuchu, Tokyo 183-8509, Japan; 6 Division of Rare Cancer Research, National Cancer Center Research Institute, 5-1-1 Tsukiji, Chuo-Ku, Tokyo 104-0045, Japan

**Keywords:** angiosarcoma, Dog, hemangiosarcoma, primary culture, transcriptional analysis, *PLAAT3*

## Abstract

Canine hemangiosarcoma (HSA) is a highly aggressive vascular malignancy encountered in veterinary practice and exhibits histopathological and molecular similarities to human angiosarcoma (AS). Despite its clinical importance, the molecular mechanisms driving canine HSA remain poorly understood, limiting the development of effective therapeutic approaches. To address this challenge, we established patient-derived canine HSA 2.5D organoids as a preclinical model. Following validation of lineage-specific marker expression, we evaluated drug responses and performed transcriptomic analyses comparing HSA organoids with nodular hyperplasia (NH) samples to identify molecular alterations associated with malignant transformation. Differential gene expression analysis revealed several genes enriched in HSA, including Phospholipase A and Acyltransferase 3 (*PLAAT3*), which was significantly upregulated in HSA organoids. Functional studies demonstrated that both siRNA-mediated silencing and pharmacological inhibition of *PLAAT3* markedly reduced the invasive capacity of HSA organoid cells while exerting only modest effects on cell proliferation. In addition, orthotopic implantation of HSA 2.5D organoids into the spleens of immunodeficient mice generated xenograft tumors with metastatic behavior and histopathological features closely resembling those of the original canine tumors. Collectively, these results establish a canine HSA 2.5D organoid model and identify *PLAAT3* as a candidate molecule associated with the invasive phenotype of canine HSA, providing a valuable platform for investigating disease biology and for future comparative studies of canine HSA and human AS.

## Simple Summary

Canine hemangiosarcoma is a highly aggressive cancer that arises from blood vessel-forming cells and often spreads rapidly to other organs. Effective treatments for this disease remain limited, partly because the biological mechanisms that drive its progression are not fully understood. In this study, we developed a laboratory model using three-dimensional tumor-like structures grown from canine hemangiosarcoma tissues collected from affected dogs. These structures retained important characteristics of the original tumors and were used to investigate drug responses and changes in gene activity associated with the disease. Our analysis identified phospholipase A and acyltransferase 3 as a molecule that was more abundant in hemangiosarcoma than in non-cancerous tissue. Reducing the activity of this molecule, either by suppressing its production or by using a chemical inhibitor, markedly decreased the ability of cancer cells to invade surrounding areas, suggesting that it may contribute to tumor progression. The tumor model also reproduced tumor growth and spread when implanted into mice. These findings provide a useful experimental model for studying canine hemangiosarcoma and identify phospholipase A and acyltransferase 3 as a candidate molecule associated with the invasive phenotype of canine HSA. Because canine hemangiosarcoma shares important features with human angiosarcoma, this model may also provide a useful platform for future comparative studies of these diseases.

## 1. Introduction

Canine hemangiosarcoma (HSA) is a malignant tumor, which accounts for a relatively low percentage (1.3–2.8%) of canine cancer cases [1]. As a highly invasive cancer, HSA often metastasizes to the liver and lungs through blood vessels [2,3]. Because HSA shares several pathological characteristics and molecular signatures with human angiosarcoma (AS) [4], it is considered a more relevant preclinical model than rodent models for advancing AS research.

Conventional therapies for HSA include surgery and chemotherapy with drugs such as doxorubicin. However, the median survival time after chemotherapy is six months, whereas that after surgery alone is two to three months [5]. Therefore, to better understand disease progression and develop new treatment options, a more suitable animal model is required to uncover the transcriptional profiles associated with cancer progression.

Clinically, splenic masses in dogs are mostly benign tumors, including nodular hyperplasia (NH). Splenic NH is defined as the overgrowth of normal splenic components, forming a well-restricted proliferative lesion [6]. During new target development, a common method includes the comparison of the transcriptional patterns between cancerous and healthy tissues. Several sequencing studies comparing HSA with normal healthy tissues have reported various mutated genes such as *TP53* and *PIK3CA*, which are implicated in DNA repair, and those associated with the PI3K/AKT/mTOR and MAPK/ERK pathways, which are involved in cell proliferation [7,8,9]. However, a comparison of HSA with healthy tissues may not completely reveal the characteristics of cancerous proliferation. Fast-proliferating NH may provide a reference for identifying the cause of malignant metastasis. Although the propensity for metastasis has been reported to be highly associated with lysosomal [10] and membrane dysfunction [11], their roles in HSA remain unclear.

Several currently established mouse models replicating the biological and molecular characteristics of HSA have been reported; however, these models are mainly based on the direct subcutaneous transplantation of patient tumor tissues into immunodeficient mice [12,13]. Therefore, these models cannot recapture the metastatic features of the original tumor within the liver or other organs. Given that high invasion and metastasis are key factors responsible for treatment failure and poor prognosis in dogs with HSA, a new reliable mouse model is urgently needed for mechanistic studies and drug development.

In previous studies, we established a 2.5D organoid culture method using tumor tissues from dogs with bladder, breast, and lung cancers, which could recapitulate the characteristics of the original tumor tissues [14,15]. In this exploratory study, we aimed to establish and characterize HSA 2.5D organoids and compare them with NH organoids to investigate their biological and transcriptomic characteristics. Tumorigenesis ability of generated HSA 2.5D organoids was tested via splenic injection. HSA markers CD31, vWF, and VEGF were validated, and gene expression profiles between HSA and NH organoids were examined along with transcriptional differences, particularly those related to metastasis and invasion, such as lysosomal membrane-related pathways and genes such as phospholipase A and acyltransferase (*PLAAT)3*. Additionally, the effects of *PLAAT3* knockdown and chemical inhibition in HSA cells on their invasion and proliferation were demonstrated.

## 2. Materials and Methods

### 2.1. Sample Collection and HSA 2.5D Organoid Culture

All dog owners provided written informed consent for the present study, and all experimental procedures were carried out according to the guidelines of the Institutional Animal Care and Use Committee of Tokyo University of Agriculture and Technology (Approval number: 0020007; approval date: 6 May 2021). Sample information is presented in Table 1. Standard hepatectomy and/or splenectomy and mastectomy were performed with informed consent from the owner. Canine tissue samples used in this study were obtained from surplus tissue collected during routine surgical procedures performed for clinical diagnosis or treatment. No anesthesia or euthanasia was performed specifically for the purposes of this study. Tissue sections used for primary culture were obtained from the same positions that were used in pathological diagnosis and immunohistochemistry (IHC) staining.

The samples were immediately transferred to a cooled shipping medium and transported to our laboratory. 2.5D organoids were generated using methods described in our previous study [14]. Briefly, after receipt from the clinic, the tumor tissue samples were minced and digested with Liberase™ TH (Roche Diagnostics, Mannheim, Germany) for 20 min at 37 °C and then centrifuged at 600× *g* for 3 min. The tissue pellets were trypsinized for 3 min, and the digested cells were collected using a cell strainer. Red blood cells were then removed using RBC lysis buffer. After washing three times with PBS, the primary cells were seeded in dishes containing our prepared 2.5D organoid medium.

Culture conditions, medium composition, supplements, growth factors, cell handling, and passaging procedures were identical to those described in our previous study. The culture medium comprised advanced DMEM including 50% Wnt, R-spondin, and Noggin conditioned medium; 100 µg/mL Primocin; 10 mM nicotinamide; 1% GlutaMax; and 1 mM N-Acetyl-L-cysteine (Thermo Fisher Scientific, Waltham, MA, USA); 500 nM A83-01 (Adooq Bioscience, Irvine, CA, USA); and 50 ng/mL mouse EGF (PeproTech, Rocky Hill, NJ, USA). All representative phase-contrast images of the cultured cells were captured using a light microscope (BX-52; Olympus, Tokyo, Japan).

### 2.2. Hematoxylin and Eosin (H&E) Staining

Paraffin sectioning and H&E staining were conducted according to standard procedures, as described previously [16]. All sections were stained with hematoxylin and eosin for 4 min and 30 s, respectively. Sections were observed and imaged using a light microscope (BX-52; Olympus, Tokyo, Japan).

### 2.3. Immunohistochemical (IHC) Staining

IHC staining was performed on paraffin-embedded sections, 4 μm in thickness. The following antibodies were used: rabbit polyclonal CD31 antibody (GeneTex, catalog No. GTX130274; 1:500), mouse monoclonal PLA2G16 (PLAAT3) antibody (OriGene, catalog No. TA506908S; 1:200), rabbit polyclonal Von Willebrand factor antibody (abcam, ab6994; 1:200), and anti-VEGF polyclonal antibody (Bioss, catalog No. bs-1665R; 1:200). Sections were deparaffinized with xylene and subsequently rehydrated with ethanol and distilled water. For antigen retrieval, sections were autoclaved at 121 °C with sodium citrate buffer for 5 min, followed by cooling at room temperature (RT). After washing, sections were quenched with 1% hydrogen peroxide for 30 min. Sections were incubated overnight at 4 °C with primary antibodies (CD31, 1:500; PLAAT3, 1:200; vWF, 1:200; and VEGF, 1:200) diluted in 0.5% casein in a humid box. After washing with PBS, sections were incubated in an HRP system and stained using a Dako kit (K3468) following the manufacturer’s protocol. After DAB staining, sections were counterstained with hematoxylin, dehydrated using ethanol and xylene, mounted with Polymount, dried, and examined. The sections were observed and imaged using a light microscope (BX-52; Olympus, Tokyo, Japan).

### 2.4. Immunofluorescence (IF) Staining

Immunofluorescence staining of HSA 2.5D organoids was performed as previously described [14,15]. Immunofluorescent HSA 2.5D organoid cells (2 × 10^5^/well) were seeded onto round cover glasses in a 6-well plate. After reaching 80% confluence, cells were fixed in 4% PFA for 1 h at RT. Subsequently, the cover glasses were washed three times with PBS and blocked with 1.5% normal goat serum (diluted in PBS) for 30 min at RT, and subsequently incubated in a humid box overnight at 4 °C with primary antibodies (CD31, 1:300; PLAAT3, 1:100; vWF, 1:200; and VEGF, 1:200) diluted in PBS. After washing with PBS, the slides were incubated with Hoechst (1:1000) and with a secondary antibody for 60 min at RT in the dark. Representative images were captured and analyzed. The secondary antibodies used were: Goat Anti-Rabbit IgG H&L (Alexa Fluor^®^ 488) (ab150077) and Goat Anti-Mouse IgG H&L (Alexa Fluor^®^ 488) (ab150113). All samples were observed under a fluorescence microscope (DAPI and FITC). Semi-quantitative analysis of fluorescence was performed using the ImageJ software, version 1.54r.

### 2.5. Cell Viability Screening

Cytotoxicity activities were evaluated via the PrestoBlue™ Cell Viability assay. Cells (1 × 10^4^ cells/well) were seeded in 96-well plates containing 2.5D medium. After 24 h, cells were treated with dimethyl sulfoxide (DMSO; vehicle), toceranib (Sigma Aldrich, America, MI, USA; at 2 μM, 4 μM, and 8 μM), carboplatin (FUJIFILM Wako Pure Chemical, Tokyo, Japan; at 1 μM, 10 μM, and 100 μM), cyclophosphamide (at 1 μM, 10 μM, and 100 μM), or doxorubicin (Cayman Chemical, Ann Arbor, MI, USA; at 1 μM, 10 μM, and 100 μM), and incubated at 37 °C for 72 h. Following incubation, 10 μM of PrestoBlue™ Cell Viability Reagent (Thermo Fisher Scientific) was added to each well, and the cells were incubated for an additional 3 h at 37 °C. Fluorescence (emission wavelength: 585 nm) was measured using a microplate reader (TECAN, Seestrasse, Switzerland). The chemical and company names were as follows: LEI110 (MCE, HY-125254), toceranib (Sigma, No. PZ0338), doxorubicin (Cayman Chemical, 25316-40-9), cyclophosphamide (TCI, 6055-19-2), and carboplatin (Fujifilm, 41575-94-4). IC_50_ values were determined by nonlinear regression analysis of the dose–response curves using GraphPad Prism. When 50% inhibition was not reached within the tested concentration range, the IC_50_ was reported as greater than the highest concentration tested.

### 2.6. RNA Isolation and Real-Time Polymerase Chain Reaction (PCR)

Isolated RNA was converted to first-strand cDNA using the ReverTra Ace qPCR RT Kit (Toyobo Co., Ltd., Osaka, Japan) following the manufacturer’s protocol. Real-time PCR was performed using the QuantiTect SYBR I kit (Qiagen, Hilden, The Netherlands) and StepOnePlus Real-Time PCR system (Applied Biosystems, Waltham, MA, USA). The ^ΔΔ^Cq method was used to quantify the data. Each cDNA sample was amplified with primers and run in triplicate. The expression level of each gene was normalized to *GAPDH* levels in each sample. Primers (FASMAC Co., Ltd., Kanagawa, Japan) used for canine genes *MARVELD3*, *Huntingtin-interacting protein 1 (HIP1)*, *PLAAT3*, and *GAPDH* are listed in Table 2.

### 2.7. RNA Sequencing

RNA sequencing and bioinformatic analyses were performed as previously described with minor modifications [17]. Ten biologically independent original tissue samples (HSA, *n* = 5; NH, *n* = 5) were collected from the clinic and used to establish 2.5D organoid lines for subsequent RNA sequencing. The sample IDs and corresponding sample information are provided in Supplementary Table S1. Total RNA was extracted from harvested primary cultured 2.5D organoids using the FavorPrep Tissue Total RNA Purification Mini Kit (Favorgen Biotech Corp., Pingtung, Taiwan) following the manufacturer’s protocols. One microgram of total RNA from each sample was used for cDNA library preparation following poly(A) mRNA enrichment. Libraries were sequenced on an Illumina NovaSeq 6000 platform using a 150 bp paired-end (PE) strategy.

The reads were mapped to the reference genome (UU_Cfam_GSD_1.0; NCBI RefSeq assembly GCF_011100685.1) using Hisat2 (v2.2.1) [18]. Gene expression levels were quantified using HTSeq (v0.6.1) [19]. Differential expression analysis was performed using DESeq2 Bioconductor package [20]. Genes with an adjusted *p* value (Padj) ≤ 0.05 were considered differentially expressed. Principal component analysis (PCA) and hierarchical clustering were performed to assess the overall transcriptomic relationships among the HSA and NH samples. The PCA and hierarchical clustering results are shown in Supplementary Figures S1 and S2, respectively. Gene Ontology enrichment analysis was performed using GOSeq (v1.34.1) [21]. Gene set enrichment analysis (GSEA) was performed using GSEA software 4.4.0 developed by Broad Institute, Inc. [22,23] to identify significantly enriched biological pathways. Significantly enriched pathways were identified based on the normalized enrichment score (NES), nominal *p* value and false discovery rate (FDR). Gene sets with FDR q-values < 0.25 were considered significantly enriched.

### 2.8. Orthotopic Patient-Derived Xenograft Mouse Model

Ten-week-old C.B-17/IcrHsd-Prkdc scid mice (SANKYO Laboratory, Tokyo, Japan) were anesthetized via isoflurane inhalation and positioned on their right side. The area above the spleen was shaved, and the skin was sterilized using 80% ethanol. A subcostal incision (approximately 2 cm) was made below the ribs to access the peritoneal cavity, exposing the spleen. Primary cultured cells (BS24028, 1 × 10^6^ cells in 50 µL PBS per mouse) were gently resuspended and slowly injected into the spleen. Following injection, the site was gently compressed for 2 min using a cotton swab to prevent backflow. The spleen was then repositioned at its site in the abdominal cavity, and the peritoneum and skin layers were sutured. Ten weeks post-injection, mice were euthanized by intravenous administration of an overdose of pentobarbital sodium, and tumor-bearing organs were subsequently harvested and examined. All procedures were conducted with the approval of the Tokyo University of Agriculture and Technology Animal Care and Use Committee and Ethics Committee (approval number: R5-124). All procedures were done in accordance with the ARRIVE (Animal Research: Reporting of In Vivo Experiments) guidelines.

### 2.9. Cell Proliferation Assay

HSA 2.5D organoid cells were seeded in 96-well plates at 1000 cells/100 µL medium/well. The number of living cells was detected via the PrestoBlue™ Cell Viability assay on days 1, 4, and 7, and analyzed by measuring the fluorescent intensity in each well using a Tecan microplate reader, as previously described [14].

### 2.10. Boyden Chamber Invasion Assay

Matrigel was diluted with serum-free medium (advanced DMEM) at a ratio of 1:1. For invasion assays, 50 μL of diluted gel was dropped in each Transwell^®^ insert (8 μm PET membrane, Corning 3464). The gel was then solidified at 37 °C in a CO_2_ incubator for 1 h. After fixation, the extra medium was aspirated into the insert. Cells were trypsinized and resuspended in serum-free DMEM. Next, 3 × 10^4^ cells were resuspended in 300 μL serum-free media with treatment or vehicle and seeded into the upper chamber. Thereafter, 600 μL of culture medium with 10% FBS as a chemoattractant was added to the lower chambers. Cells were maintained overnight in an incubator at 37 °C with 5% CO_2_. After 24 h, the Transwell inserts were washed twice with PBS, and the cells were fixed with 4% PFA for 2 min and methanol for 20 min at RT. The cells on the upper side of the inserts were removed using cotton swabs, and the lower side of the membrane was stained with Giemsa solution for 15 min at RT. Invading and migrating cells were observed and imaged using an optical microscope (BX52, Olympus, Japan).

### 2.11. PLAAT3 siRNA Transfection

HSA 2.5D organoid cells were seeded at a density of 10^5^ cells/well in a 6-well plate. After 24 h of culture, cells were transfected with siRNA against *PLAAT3* or negative control siRNA (Sequence (5′–3′): si 1, AGCACAUCACACAACAUUA and si 2, AAUGGUGGUUAUUCAGUUC) using Lipofectamine RNAiMAX (Invitrogen) following the manufacturer’s protocol. After 72 h, the cells were harvested, and evaluations were performed via cell proliferation assay, cell invasion assay, and quantitative RT-PCR.

### 2.12. Statistical Analysis

All statistical analyses in this study were performed using GraphPad Prism (11.0.1). Data are shown as mean ± S.E.M. Comparisons between two groups were performed using unpaired Student’s *t*-test. For multiple group comparisons, one-way ANOVA followed by Dunnett’s multiple comparisons test was used. For experiments involving two independent variables, two-way ANOVA followed by Dunnett’s multiple comparisons test was applied. *p* values < 0.05 were considered statistically significant.

## 3. Results

### 3.1. Pathological Comparison of Patient Tissues with HSA and NH

**Figure 1:**
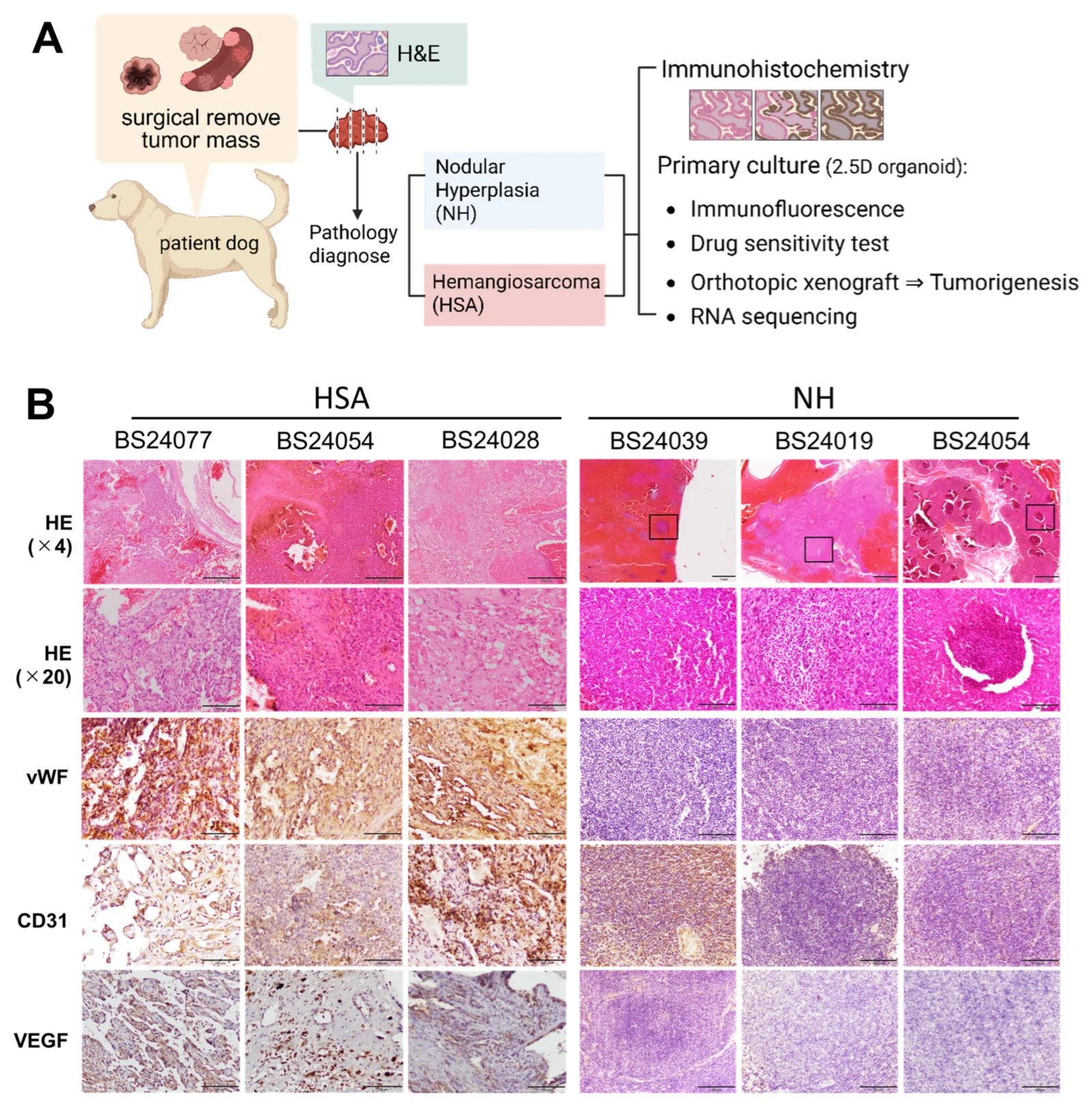
Histopathology features of hemangiosarcoma (HSA) and nodular hyperplasia (NH). The schema of the study procedure was created using Biorender (**A**). Histopathology and expression of vascular endothelial markers in HSA and NH tissues (**B**). The five representative cases of NH and HSA tissues were diagnosed via hematoxylin and eosin (H&E) staining and stained with vascular endothelial markers (CD31, VEGF, and vWF). Scale bar for H&E staining (×4) = 500 µm, all other scale bars = 100 µm.

Primary cultured 2.5D organoids were established from canine hemangiosarcoma (HSA) and nodular hyperplasia (NH) tissue following pathological diagnosis (Figure 1A,B). Histopathological examination of the original canine HSA samples revealed irregular vascular channels and atypical cells with enlarged hyperchromatic nuclei, whereas NH exhibited comparatively well-organized splenic structures with minimal atypia. IHC staining demonstrated expression of vWF, CD31 and VEGF in both HSA and NH tissues (Figure 1B). Stronger CD31 and vWF immunoreactivity was observed in HSA tissues than NH lesions. In HSA samples, CD31 staining was associated with irregular vascular-like structures and showed heterogeneous distribution within tumor regions. vWF staining varied among HSA cases, ranging from focal positive staining to weak or nearly absent signals in highly cellular areas. In contrast, NH lesions showed limited or weak staining of all three markers, primarily associated with residual vascular structures. VEGF immunoreactivity was observed in HSA samples, whereas NH tissues exhibited only weak or minimal staining.

### 3.2. Generation and Drug Sensitivity Test of Patient-Derived HSA 2.5D Organoids

**Figure 2:**
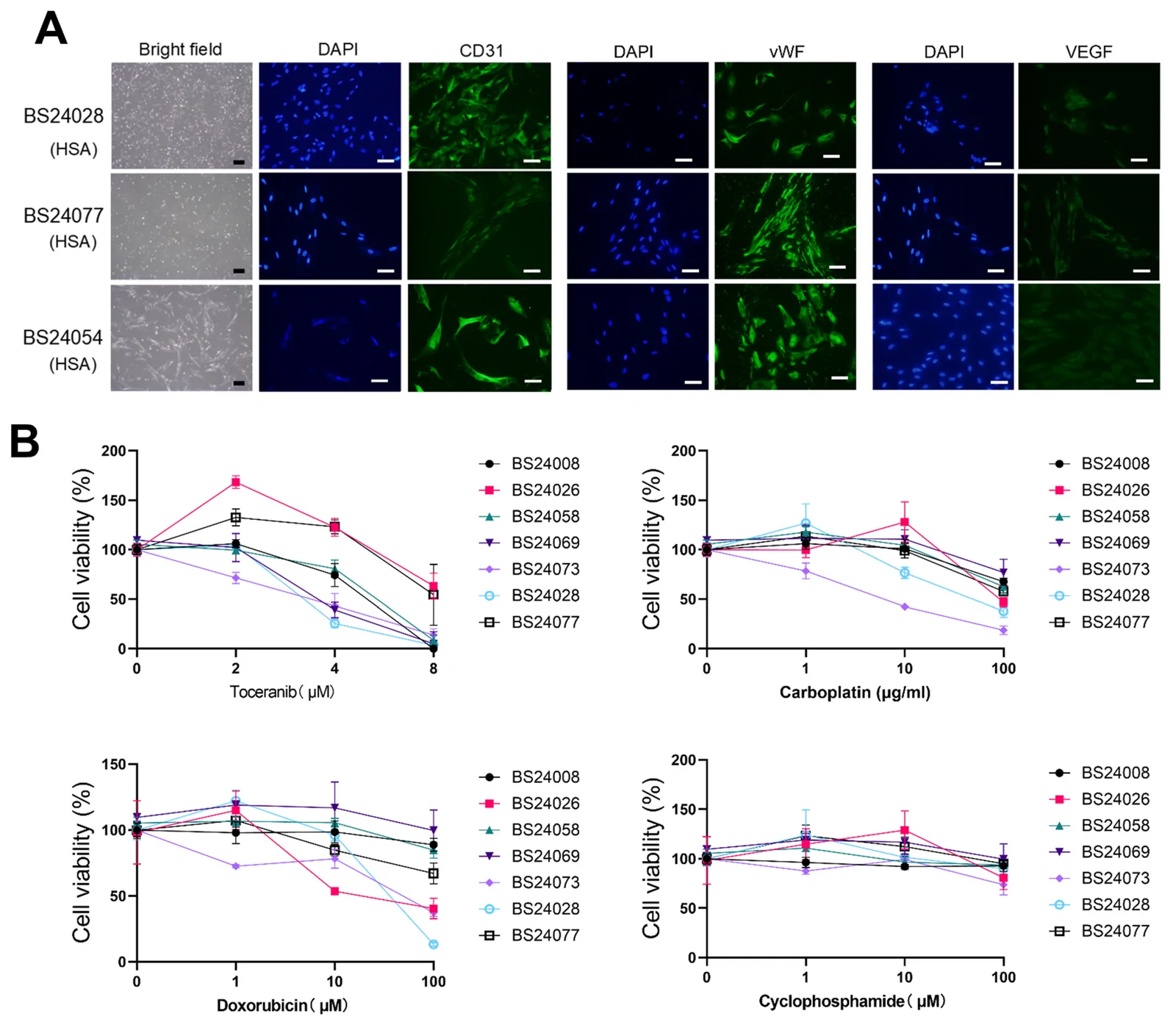
Generation and drug sensitivity tests of 2.5D organoids from dogs with hemangiosarcoma (HSA) and nodular hyperplasia (NH). Expression of vascular endothelial markers in 2.5D organoids of HSA and NH (**A**). After generating 2.5D organoids from each dog patient with HSA and NH, cells were incubated with CD31, vWF, and VEGF antibodies for immunofluorescence (IF) staining. Scale bar = 50 µm. Bright-field photos are shown. Scale bar = 200 µm. HSA 2.5D organoid cells from each dog patient (BS24008, BS24026, BS24058, BS24069, BS24073, BS24028 and BS24077) were treated with toceranib, carboplatin, cyclophosphamide, or doxorubicin at gradient concentrations, and cell viability was evaluated via the PrestoBlue™ cell viability assay (**B**). Data are expressed as mean ± S.E.M. Drug screening tests were performed using HSA 2.5D organoid cells. The IC_50_ values were calculated and summarized in Supplementary Table S2.

Primary cultured HSA 2.5D organoids showed expression patterns of the three markers similar to those observed in the original tissues (Figure 2A). Carboplatin, doxorubicin, toceranib, and cyclophosphamide are commonly used chemotherapies for HSA in pet clinics. Therefore, we conducted drug sensitivity tests using HSA 2.5D organoids to evaluate their in vitro responses to these clinically relevant drugs.

We treated HSA organoid lines with toceranib, carboplatin, cyclophosphamide and doxorubicin (Figure 2B). HSA 2.5D organoids derived from different patients exhibited variable drug sensitivities with toceranib, carboplatin and doxorubicin, suggesting interpatient heterogeneity in drug response.

### 3.3. In Vivo Tumorigenicity of HSA 2.5D Organoids in an Orthotopic Xenograft Model

**Figure 3:**
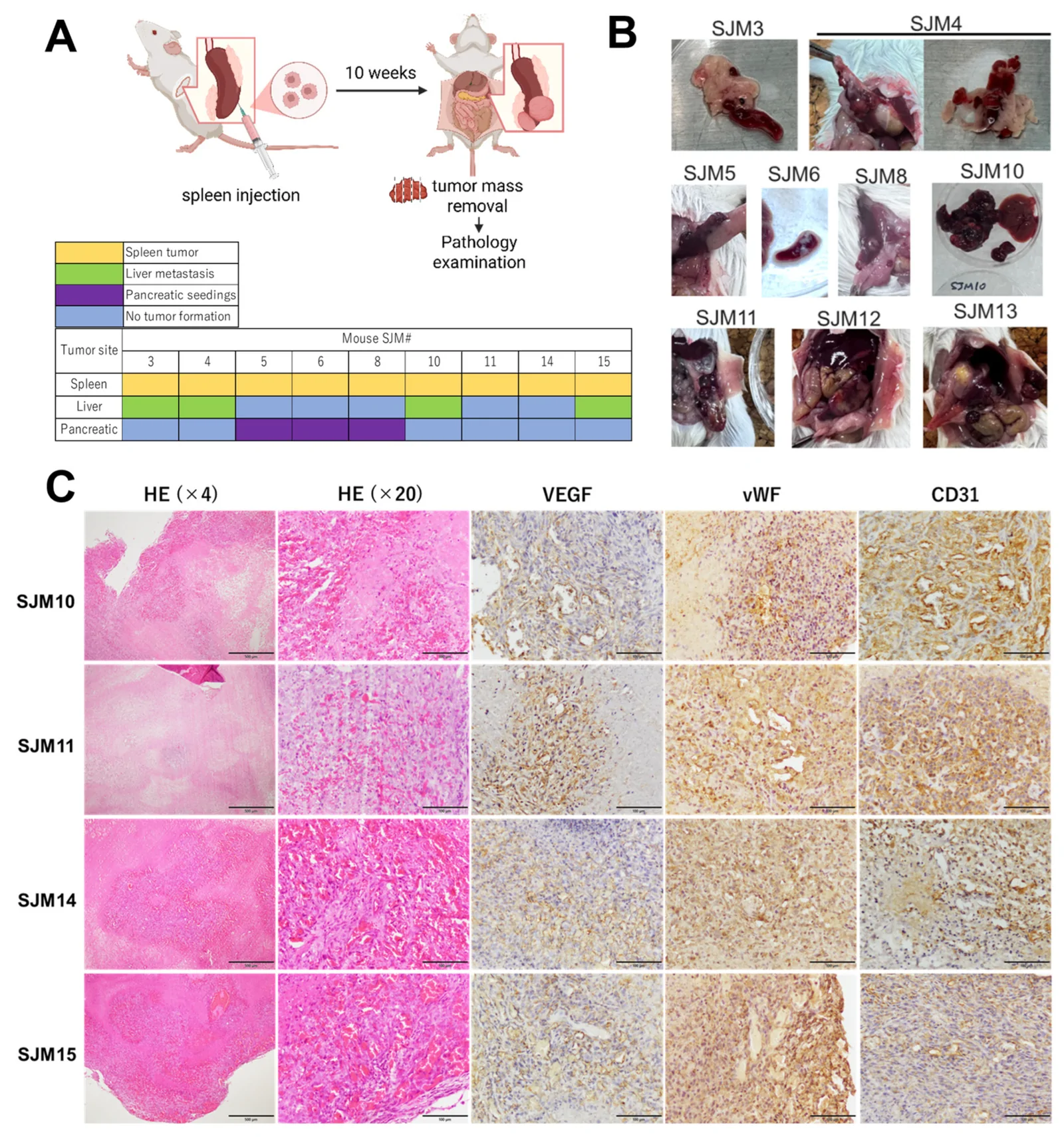
Tumorigenesis experiment via orthotopic xenograft mouse model using canine HSA 2.5D organoids. The schema of the study procedure was created using Biorender (**A**). Following 10 weeks of 2.5D HSA organoid injection into the spleens of immunodeficient mice, each organ was harvested and subjected to histopathological analysis. The occurrence of primary and metastatic tumors in orthotopic xenografts of HSA 2.5D organoids is shown. HSA formation is depicted in yellow. The green and purple boxes indicate liver metastasis and pancreatic seeding, respectively. Light blue indicates no tumor formation in a specific position. Tumor mass photos are shown (**B**). Expression of vascular endothelial markers (VEGF, vWF and CD31) in orthotopic xenograft tumor tissues (**C**). Scale bar for H&E staining (×4) = 500 µm, all other scale bars = 100 µm.

Next, to assess whether HSA 2.5D organoids retained tumorigenic potential in vivo, an orthotopic xenograft model was established by splenic injection (Figure 3A). Ten weeks after splenic injection with HSA 2.5D organoid cells (BS24028) in C.B-17/IcrHsd-Prkdc SCID mice, orthotopic splenic HSA tumors developed in 9/9 mice. Among these mice, liver metastasis was observed in 4 of 9 mice, consistent with the metastatic lesion observed in the original canine patient, while pancreatic seeding was observed in 3 mice (Figure 3B).

Histopathological examination using H&E staining revealed abnormal cleft-like structures in xenografted splenic tissues, which morphologically resembled those in the original canine HSA tumor tissue. Enlarged nuclei and extensive infiltrative proliferation of atypical cells were observed inside and outside the spleen lesions (Figure 3C).

Additionally, IHC staining demonstrated expression of VEGF, vWF and CD31 in xenografted tumor tissues (Figure 3C). These findings support that the primary cultured 2.5D HSA organoids retained tumorigenic capacity and several histopathological features associated with canine HSA in vivo.

### 3.4. Comparison of Transcriptional Profiles Between HSA and NH 2.5D Organoids

**Figure 4:**
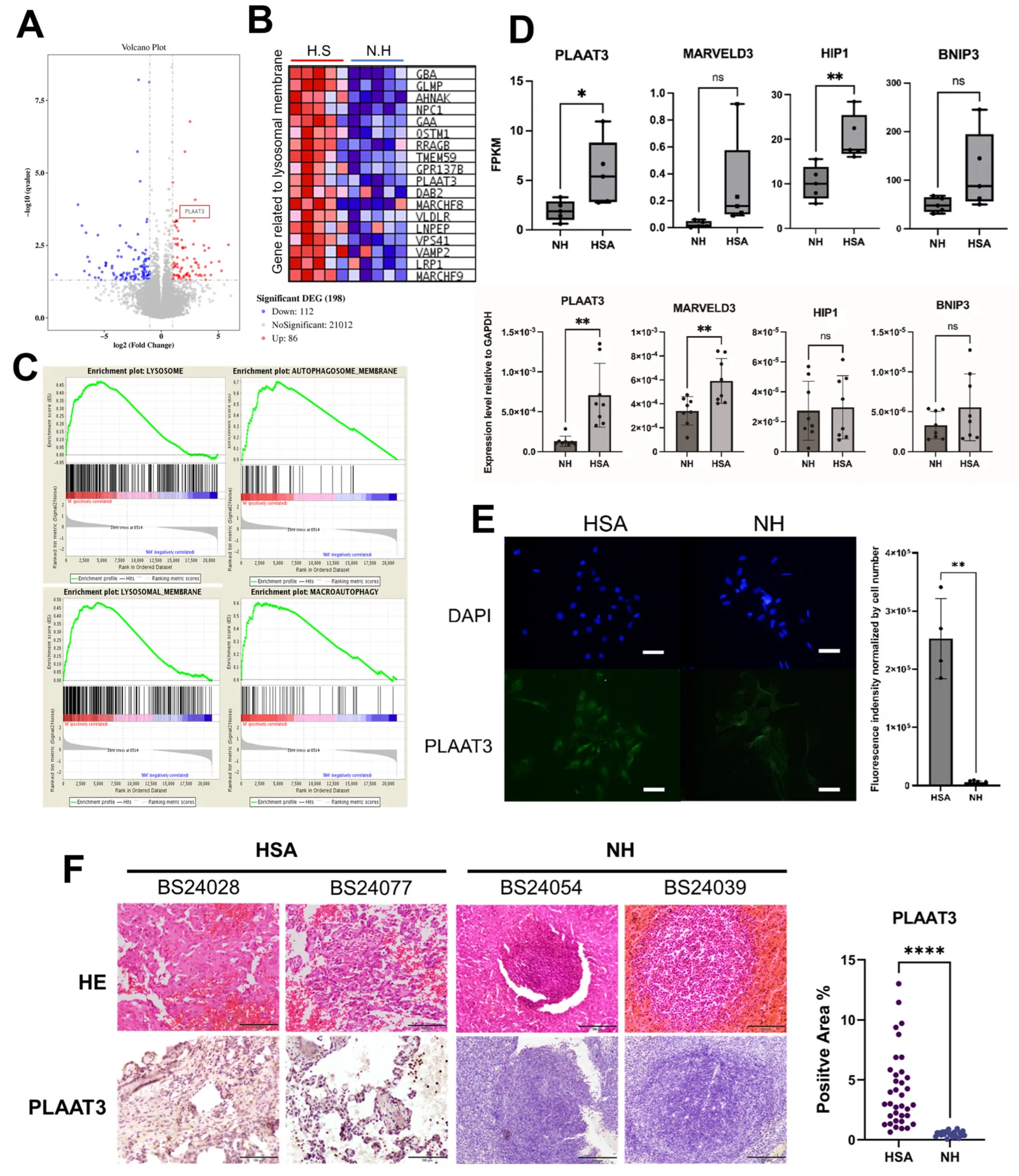
Comparison of transcriptional profiles between HSA and NH 2.5D organoids. Transcriptomic analysis experiments were performed on NH and HSA 2.5D organoids (**A**). Comparison of gene expression between NH and HSA 2.5D organoids presented as a volcano plot. Upregulated genes are shown in red, whereas downregulated genes are in blue. (**B**) Lysosomal membrane-related gene expression between NH and HSA 2.5D organoids; red typically indicates high or upregulated expression, while blue indicates low or downregulated expression. (**C**) Gene set enrichment analysis (GSEA) using RNA-sequencing data and normalized enrichment scores. (**D**) FPKM scores of specific regulated genes and corresponding quantitative real-time PCR results. Five biologically independent organoid lines were analyzed per group (HSA, *n* = 5; NH, *n* = 5), and each data point represents one independent organoid line. Data are expressed as mean ± S.E.M. ∗ *p* < 0.05 vs. NH, ∗∗ *p* < 0.01 vs. NH. *PLAAT3* expression was compared between HSA and NH 2.5D organoids using IF staining (NH: BS24019, HSA: BS24028) (**E**). IF staining was independently performed four times using separately cultured samples from each organoid line. Data are expressed as mean ± S.E.M. of four independent experiments. Scale bar = 100 µm.∗∗ *p* < 0.01 vs. NH. *PLAAT3* expression was compared between HSA and NH tissues using immunohistochemistry (**F**). Scale bar = 100 µm. HSA (*n* = 36), NH (*n* = 21), ∗∗∗∗ *p* < 0.0001, unpaired *t*-test with Welch’s correction. ns: not significant.

RNA sequencing was conducted to compare transcription profiles between NH and HSA 2.5D organoids (Figure 4A,B). PCA and hierarchical clustering were performed to visualize the overall transcriptomic relationships among the HSA and NH samples (Supplementary Figures S1 and S2). Gene set enrichment analysis of differentially expressed genes (DEGs) revealed enrichment of pathways associated with autophagy, autophagic membrane, lysosomes, and lysosomal membrane in HSA 2.5D organoids (Figure 4C).

Genes related to the cellular membrane, lysosomal membrane, and autophagy, including *HIP1*, *PLAAT3*, and *BNIP3*, were found upregulated in HSA 2.5D organoids compared to NH organoids (Figure 4D). Additionally, quantitative real-time PCR analysis results further supported the increased expression of *PLAAT3* in HSA 2.5D organoids. In addition, IF staining demonstrated increased PLAAT3 expression in HSA 2.5D organoids (Figure 4E). IHC staining and quantification of PLAAT3 in HSA and NH tissues are also shown in Figure 4F.

Among the upregulated genes identified by transcriptomic analysis, *PLAAT3* was selected for further investigation because of its consistently elevated expression across multiple analyses. Given its reported association with membrane remodeling, *PLAAT3* was considered a potential candidate involved in HSA progression.

### 3.5. Effects of PLAAT3 Inhibition on Cell Proliferation and Invasion in HSA 2.5D Organoids

**Figure 5:**
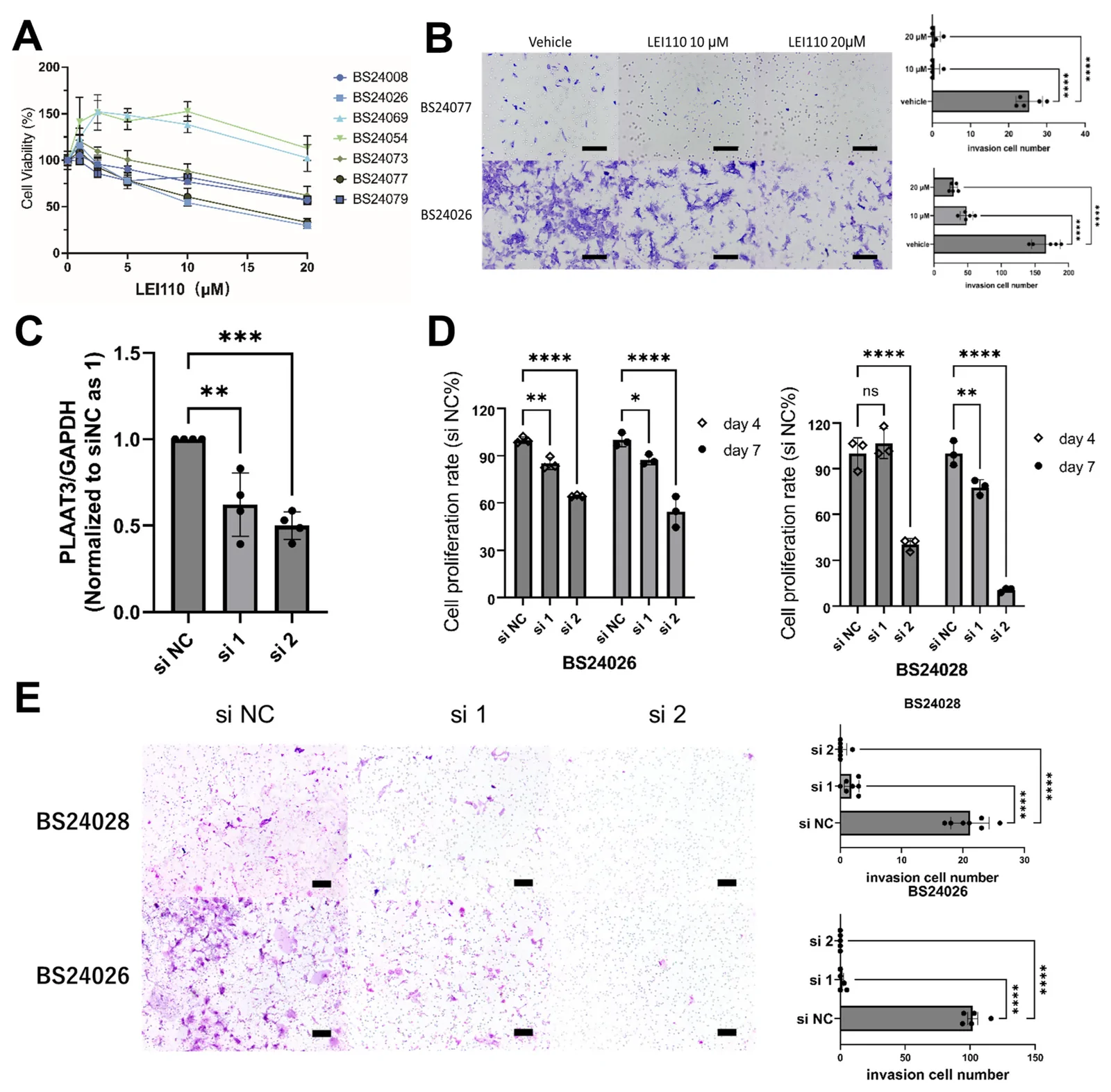
Effects of *PLAAT3* inhibition on cell proliferation and invasion of HSA 2.5D organoids. The effects of the PLAAT3 inhibitor LEI110 on the cell viability of HSA 2.5D organoids were evaluated via the PrestoBlue™ Cell Viability assay (**A**). IC_50_ > 20 μM in all strains, as 50% inhibition was not reached at the highest concentration tested (20 μM). The effects of LEI110 on the cell invasion of HSA 2.5D organoids via the cell invasion assay (**B**). Results are expressed as mean ± S.E.M., *n* = 6. ∗∗∗∗ *p* < 0.0001 vs. Vehicle. The efficacy of *PLAAT3* gene knockdown (**C**). Results are expressed as mean ± S.E.M., ∗∗ *p* < 0.01 vs. Si NC, ∗∗∗ *p* < 0.001 vs. Si NC. The effects of *PLAAT3* gene knockdown on the proliferation (at day 4 and 7 after cell seeding) of HSA 2.5D organoids (BS24026 and BS24028) were evaluated via cell proliferation assay. (**D**). Results are expressed as mean ± S.E.M., *n* = 6. ∗ *p* < 0.05 vs. si NC, ∗∗ *p* < 0.01 vs. si NC, ∗∗∗∗ *p* < 0.0001 vs. si NC. The effects of *PLAAT3* gene knockdown on the cell invasion of HSA 2.5D organoids (**E**). Scale bar = 100 µm. Results are expressed as mean ± S.E.M., ∗∗∗∗ *p* < 0.0001 vs. Si NC.

To further investigate the functional role of PLAAT3 in HSA, pharmacological inhibition was performed using LEI110, and its effects on cell proliferation and invasion in HSA 2.5D organoids were evaluated. LEI110 exhibited varying inhibitory effects on several HSA 2.5D organoid lines (Figure 5A). In the invasion assay, high-dose treatment (10 and 20 μM) of LEI110 significantly reduced the number of invasive cells compared with vehicle-treated controls, suggesting *PLAAT3* inhibition may be associated with reduced invasive capacity in HSA 2.5D organoids (Figure 5B).

To further validate the effects of *PLAAT3* inhibition, we subsequently performed genetic knockdown using siRNA (Figure 5C). Cell viability assay showed a modest inhibitory effect in knockdown cells on day 4 and a significant inhibitory effect on day 7 in each cell line (Figure 5D). In addition, *PLAAT3* knockdown remarkably reduced invasive activity compared with negative control siRNA treatment (Figure 5E).

## 4. Discussion

CD31, CD34, and vWF are core endothelial markers commonly investigated in AS and HSA research [24,25], and are valuable in AS diagnosis to confirm endothelial cell origin. Active VEGF expression is observed in canine vascular tumors [26] such as HSA, as well as in breast cancers [27,28]. Although weak staining was occasionally observed in NH lesions, CD31 and vWF immunoreactivity tended to be more heterogeneous and irregular in HSA tissues. The heterogeneous staining pattern observed in HSA may reflect variable differentiation states of neoplastic endothelial cells [29]. In particular, reduced vWF staining in highly cellular regions is consistent with previous reports describing decreased endothelial differentiation in proliferative vascular tumors [30].

2.5D HSA organoids were successfully generated from patient HSA tissues. In this study, orthotopic splenic injection successfully generated xenograft tumors from canine HSA-derived 2.5D organoids. Histopathological examination demonstrated that the xenograft tumors retained morphological characteristics of the original canine HSA tissues, including vascular-like cleft formation and marked nuclear atypia. Furthermore, immunoreactivity of xenograft tumor tissues for endothelial-associated markers, including vWF [31] and CD31 [32], as well as the angiogenesis-related marker VEGF [33], showed distribution patterns comparable to those observed in the original tumor tissues. These findings suggest that the xenograft tumors preserved key endothelial and malignant vascular characteristics of canine HSA in vivo.

Previous studies in human cancers, including colorectal cancer [34,35], renal cell carcinoma [36], and osteosarcoma [37], have demonstrated that orthotopic transplantation models reproduce clinically relevant metastatic behavior more closely than heterotopic transplanted models. In this study, liver metastases were observed in 4 out of 9 xenograft cases, suggesting partial reproduction of metastatic behavior in vivo. Compared with conventional subcutaneous xenograft models, orthotopic splenic implantation may provide a more anatomically relevant tumor environment and enable partial reproduction of metastatic behavior, including liver metastasis. However, metastatic formation was inconsistent among cases, which may be attributable to the limited observation period (10 weeks) or leakage from the injection site. Therefore, further optimization and validation are required to improve the robustness and reproducibility of this metastasis model. Several non-metastatic cases in this study showed pancreatic seeding, adjacent to the injection site, suggesting possible local tumor dissemination.

Spontaneous HSA pathology is frequently associated with the coexistence of tumor cells and infiltrating inflammatory cells, which may increase the risk of contamination during direct genetic expression detection in clinical samples. Therefore, the primary cell culture method established in the present study may provide a useful tool to address this issue.

Transcriptomic analysis revealed upregulation of lysosome-associated pathways in HSA samples, suggesting altered lysosomal activity. A previous study comparing canine hemangiosarcoma with normal granular cells showed lysosome-like ultrastructural proteinaceous accumulation within single-membrane vesicles [38], supporting lysosomal involvement in HSA. Consistent with these findings, lysosome-related genes associated with lysosomal trafficking and function, including *GLMP* and *GAA* [39,40], showed elevated expression trends, whereas *PLAAT3* expression was significantly increased in HSA samples compared with NH.

*PLAAT3*, which belongs to the PLAAT family, is known to exhibit phospholipase A2 activity, mediating lipid reprogramming and glycerophospholipid remodeling in cancer cells; *PLAAT3* is consistently reported to be highly expressed and activated in various cancers [41,42,43,44]. Previous studies have reported that *PLAAT3* is associated with membrane remodeling, cathepsin dysregulation, and increased invasive behavior in several cancers [45]. Consistent with these reports, our *PLAAT3* knockdown experiments using siRNA and pharmacological inhibition of *PLAAT3* in HSA 2.5D organoids demonstrated significant inhibition of cell invasion. Given that *PLAAT3* is related to metastasis and membrane permeability, and is associated with increased drug sensitivity in human osteosarcoma, we carried out a combination treatment cell viability test; a higher sensitivity to each drug was observed upon co-treatment with a *PLAAT3* inhibitor.

Moreover, lipid accumulation in endothelial cells is related to angiogenesis and angiogenic sprouting [46,47]. *PLAAT3* is involved in lipid accumulation within liver cells via its PLA2 activity [48]. These previous findings raise the possibility that the increased PLAAT3 expression observed in HSA may also be associated with angiogenic processes; however, this possibility was not directly investigated in the present study. Although pharmacological inhibition and knockdown of *PLAAT3* led to a mild reduction in cell proliferation, our findings primarily support an association of *PLAAT3* with cell migration and invasion rather than establishing a direct role in angiogenesis. Therefore, *PLAAT3* may contribute to the invasive phenotype of canine HSA and represents a candidate molecule warranting further investigation. However, the present study did not evaluate the association between *PLAAT3* expression and clinical outcomes or the therapeutic efficacy of *PLAAT3* targeting in vivo. Therefore, further studies incorporating clinical outcome analyses and in vivo *PLAAT3*-targeting experiments are required to determine the prognostic and therapeutic relevance of *PLAAT3* in canine HSA.

Our sequencing analysis also identified increased *HIP1* expression in HSA compared with NH. *HIP1*, which is associated with cellular trafficking [49] and tumor-related signaling pathways [50,51], has been reported previously to be up-regulated in human prostate and colon cancers [52]. Although the biological significance of *HIP1* in canine HSA remains unclear, the increased expression observed in this study suggests that further investigation may be warranted. Although transcriptome sequencing showed only a modest increase in *MARVELD3* expression in HSA compared with NH, RT-PCR analysis showed a significant increase. *MARVELD3* from TAMP family is associated with tight junction regulation and epithelial remodeling. It is found to be upregulated in several human cancers [53,54,55]. Although the precise biological roles of *HIP1* and *MARVELD3* in canine HSA is still unclear, their increased expression in HSA suggested potential involvement in HSA-associated cellular regulation. Somatic mutations in *PIK3CA* and *TP53* have been reported in both HSA and AS; however, transcript expression levels of these genes did not show significant differences from our results.

An important limitation of this study is the relatively small number of biologically independent samples. RNA-seq analysis was performed using five HSA and five NH organoid lines, and the functional and xenograft experiments were conducted using a restricted subset of the established HSA lines. Therefore, the present findings should be considered exploratory and may not fully represent the biological heterogeneity of canine HSA. Further studies using larger and independent cohorts are required to validate these findings and determine their generalizability. Another limitation is that, although canine HSA shares several pathological and molecular characteristics with human AS, a direct transcriptomic comparison with human AS datasets was not performed in the present study. Therefore, further cross-species transcriptomic analyses will be required to establish the translational relevance of the molecular findings identified here.

In conclusion, we established patient-derived HSA 2.5D organoids that retained tumorigenic potential and identified *PLAAT3* as a candidate molecule associated with HSA invasion. Although the underlying mechanisms and on-target effects require further validation, our findings provide proof-of-concept evidence supporting further investigation of *PLAAT3* in canine HSA.

## Figures and Tables

**Table 1:** Patient information.

Group	Sample No.	Age	Gender	Breed	Sample Tissue Site
HSA	BS23019	7	♂	Labrador Retriever	spleen
HSA	BS22032	12	♀	Miniature Dachshund	spleen
HSA	BS23014	11	♂	Miniature Schnauzer	spleen
HSA	BS23011	15	♂	Miniature Dachshund	spleen
HSA	BS23026	10	♂	Mixed	spleen
HSA	BS24008	11	♀	Shetland Sheepdog	spleen
HSA	BS24026	11	♂	Miniature Dachshund	pelvic lymph node
HSA	BS24028	13	♂	Scottish Sheepdog	spleen
HSA	BS24054	12	♀	Golden Doodle	spleen
HSA	BS24058	6	♂	Standard Poodle	spleen, left submandibular lymph node
HSA	BS24061	no info	♀	Mixed	spleen
HSA	BS24063	14	♂	Shiba Inu	spleen
HSA	BS24069	14	♂	Shiba Inu	spleen
HSA	BS24073	5	♀	Miniature Dachshund	spleen, skin mass
HSA	BS24077	no info	♂	Mixed	spleen
NH	BS23030	10	♀	Shiba Inu	Celiac lymph node
NH	BS23039	15	♂	Miniature Dachshund	spleen
NH	BS23033	12	♂	Maltese	spleen
NH	BS23021	11	♂	Mixed	spleen mass, spleen lymph node
NH	BS23032	8	♀	Mixed	spleen
NH	BS24054	12	♀	Golden Doodle	spleen

**Table 2:** Primers for real-time PCR analysis.

Gene Name	Forward	Reverse
Canine *MARVELD3*	CAGGGGTTACCGAAAAGTCA	TTCATCGCTCACCAACAGAG
Canine *HIP1*	CCAGCTTGCCAAAGACCAAC	GAAGTGGCAGCCATCTCCTT
Canine *PLAAT3*	ACACTGGGCCATCTACGTTG	TTGCTTCTGCCGCTTGTTTC
Canine *GAPDH*	AACTCCCTCAAGATTGTCAGCAA	CATGGATGACTTTGGCTAGAGGA

## Data Availability

The data presented in this study are openly available in the National Center for Biotechnology Information Sequence Read Archive (NCBI SRA) under BioProject ID PRJNA1399620, with BioSample accession numbers SAMN54501156, SAMN54501157, SAMN54501158, SAMN54501159, SAMN54501160, SAMN54501161, SAMN54501162, SAMN54501163, SAMN54501164, and SAMN54501165.

## Supplementary Materials

The following supporting information can be downloaded at: https://www.mdpi.com/article/10.3390/vetsci13090879/s1, Figure S1: Principal component analysis (PCA) of RNA-seq data. Figure S2: Hierarchical clustering analysis of differentially expressed genes. Table S1: Sample information for RNA sequencing. Table S2: IC_50_ values for drug screening shown in Figure 2B.
