# Supplementary material for: Modeling Canine Hemangiosarcoma Progression Using Patient-Derived 2.5D Organoids and Orthotopic Xenografts
Source: Vet Sci. 2026 Aug 27;13(9):879. doi: 10.3390/vetsci13090879 (PMC13611867; doi:10.3390/vetsci13090879)
Supplement: Supplementary file 1 [file vetsci-13-00879-s001.zip › vetsci-4486086-supplementary.pdf]

Supplementary Table S1. Sample information for RNA sequencing.

Sample ID	Diagnosis	RNA-seq group
BS23014	HSA	M
BS23019	HSA	M
BS23011	HSA	M
BS22032	HSA	M
23026(BS23026)	HSA	M
BS23032	NH	NM
BS23021	NH	NM
BS23033	NH	NM
BS23039	NH	NM
BS23030	NH	NM

M, malignant (HSA);

NM, non-malignant (NH).

Supplementary Table S2. IC_{50} values for drug screening shown in Figure 2B.

Drug	BS24008	BS24026	BS24058	BS24069	BS24073	BS24028	BS24077
Toceranib (μM)	4.23	>8	5.20	3.85	3.32	3.81	>8
Carboplatin(μg/ml)	>100	98.59	>100	>100	7.091	55.20	>100
Doxorubicin(μM)	>100	34.40	>100	>100	50.01	43.20	>100
Cyclophosphamide(μM)				>100			

Supplementary Figure S1. Principal component analysis (PCA) of RNA-seq data.

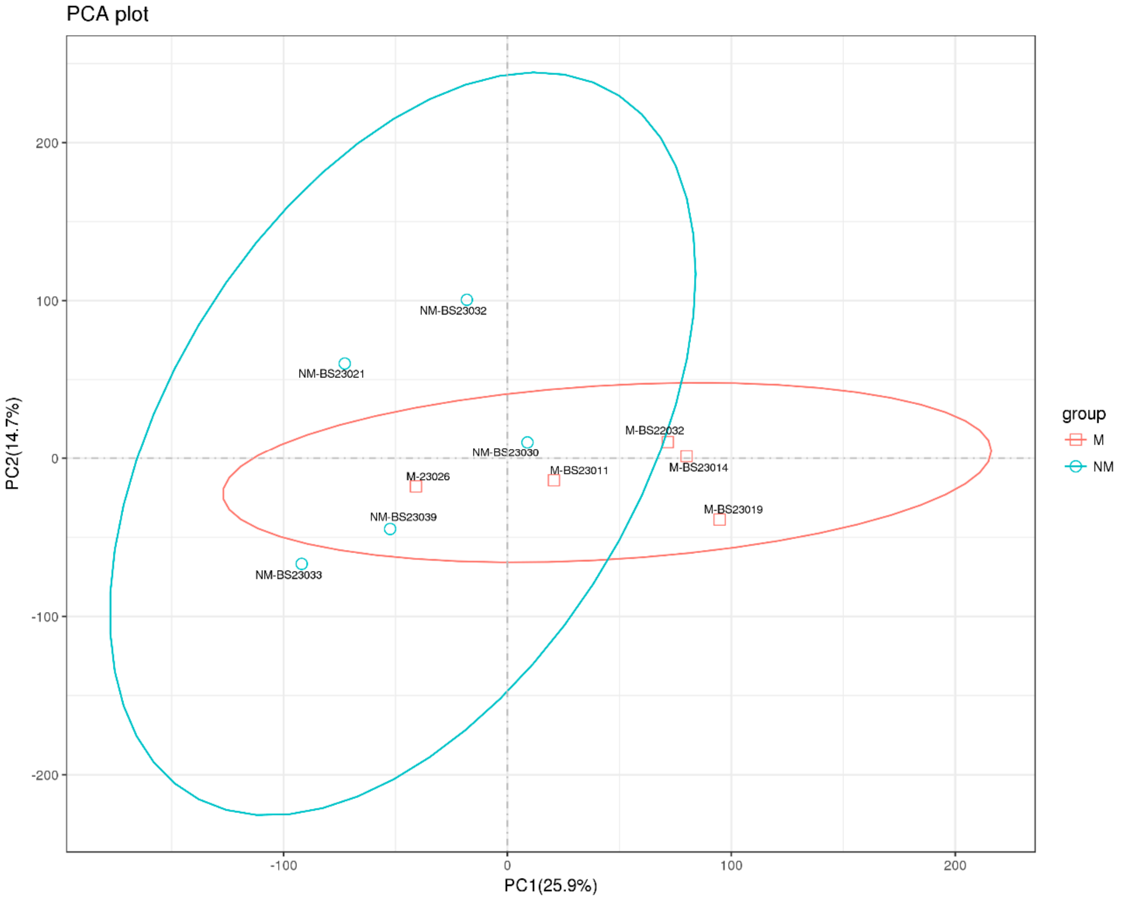

This figure shows the distribution of RNA-seq samples based on principal component analysis (PCA). Sample information is provided in Supplementary Table S1. M refers to malignant HSA samples, while NM refers to non-malignant NH samples.

Supplementary Figure S2. Hierarchical clustering analysis of differentially expressed genes.

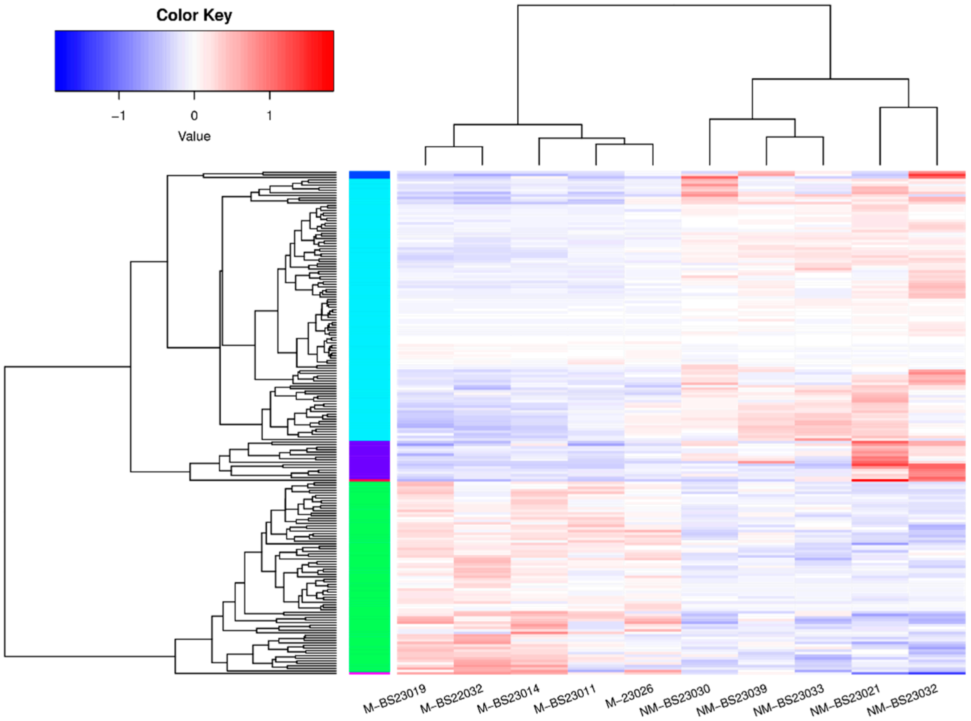

Heatmap showing the expression patterns of differentially expressed genes across HSA (M) and NH (NM) samples. Log10(FPKM + 1) values were used for clustering. Red and blue indicate relatively high and low gene expression levels, respectively.
